# Crystal structures of a series of bis­(acetyl­aceto­nato)oxovanadium(IV) complexes containing N-donor pyridyl ligands

**DOI:** 10.1107/S2056989020006246

**Published:** 2020-05-15

**Authors:** Jeffrey A. Rood, Steven R. Reehl, Kaitlyn A. Jacoby, Allen Oliver

**Affiliations:** a Elizabethtown College, Department of Chemistry and Biochemistry, 1 Alpha Drive, Elizabethtown, PA 17022-2298, USA; b University of Notre Dame, Department of Chemistry and Biochemistry, Notre Dame, IN 46556-5670, USA

**Keywords:** crystal structure, vanadium(IV), coordination compound, *cis*/*trans* isomers, bidentate ligands

## Abstract

Crystal structures for a series of bis­(acetyl­acetonato)oxovanadium(IV) complexes containing *N*-donor pyridyl ligands are reported, namely, bis­(acetyl­acetonato-κ^2^ *O*,*O*′)oxido(pyridine-κ*N*)vanadium(IV), [V(C_5_H_7_O_2_)_2_O(C_5_H_5_N)], **1**, bis­(acetyl­acetonato-κ^2^ *O*,*O*′)oxido(pyridine-4-carbo­nitrile-κ*N*)vanadium(IV), [V(C_5_H_7_O_2_)_2_O(C_6_H_4_N_2_)], **2**, and bis­(acetyl­acetonato-κ^2^ *O*,*O*′)(4-meth­oxy­pyridine-κ*N*)oxidovanadium(IV), [V(C_5_H_7_O_2_)_2_O(C_6_H_7_NO)], **3**, Compounds **1**–**3** have the formulae VO(C_5_H_7_O_2_)_2_ *L*, where *L* = pyridine (**1**), 4-cyano-pyridine (**2**), and 4-meth­oxy­pyridine (**3**). Compound **1** was previously reported [Meicheng *et al.* (1984 ▸). *Kexue Tongbao*, **29**, 759–764 and DaSilva, Spiazzi, Bortolotto & Burrow (2007). *Acta Crystallogr.*, E**63**, m2422] and redetermined here at cryogenic temperatures. Compounds **1** and **2** as pyridine and 4-cyano­pyridine adducts, respectively, crystallize as distorted octa­hedral structures with the oxo and pyridyl ligands *trans* to one another. A crystallographic twofold axis runs through the O—V—N bonds. Compound **3** containing a 4-meth­oxy­pyridine ligand crystallizes as a distorted octa­hedral structure with the oxo and pyridyl ligands *cis* to one other, removing the twofold symmetry seen in the other complexes.

## Abstract 2

Three six-coordinate complexes of bis­(acetyl­acetonato)oxovanadium(IV) containing N-donating pyridyl ligands are reported. Both *cis* and *trans* isomers were isolated and characterized from these systems.

## Chemical context   

Oxovanadium(IV) complexes have been cited as having numerous practical pharmacological applications ranging from anti­cancer agents to anti-fungal agents and, more recently, as an insulin mimetic (Singh *et al.*, 2014 ▸; Abakumova *et al.*, 2012 ▸; Amin *et al.*, 2000 ▸). Currently investigations are underway to further understand how the oxovanadium complexes perform this wide array of tasks. As an insulin mimetic, it is postulated that oxovanadium complexes inter­act with multiple points of the cell signaling pathway associated with the insulin hormone (Amin *et al.*, 2000 ▸; Srivastava & Mehdi, 2005 ▸). Alternatively, studies have shown that it inter­acts directly with glucose transporters found on the cellular surface (Hiromura *et al.* 2007 ▸; Makinen & Brady, 2002 ▸). Furthermore, vanadium has been found to have important inter­actions in DNA repair systems, which have made it a lucrative target for much oncological/pharmacological research (Abakumova *et al.*, 2012 ▸; Kostova, 2009 ▸).

Oxovanadium complexes chelated by two acetyl­acetonate ligands form a five-coordinate bonding system that can act as a Lewis acid (Nenashev *et al.* 2015 ▸; Ugone *et al.*, 2019 ▸; Costa Pessoa, 2015 ▸; Correia *et al.* 2017 ▸). This system can undergo a reaction with a Lewis base to increase its coordination bonding number to six. Of the extensive studies regarding the properties and applications of such complexes, relatively few single-crystal structures have been reported. For instance, five compounds containing N-donor ligands, a focus of this work, have been characterized by single-crystal diffraction (Meicheng *et al.*, 1983 ▸, 1984 ▸; Silva *et al.*, 2013 ▸; Kadirova *et al.*, 2009 ▸; da Silva *et al.* 2007 ▸; Caira *et al.*, 1972 ▸). Given the structural dependence on functions and application, a deeper study of the mol­ecular structure of such complexes is warranted. In this work, we describe the structures of VO(C_5_H_7_O_2_)_2_ *L*, where *L* = pyridine (**1**), 4-cyano-pyridine (**2**), and 4-meth­oxy­pyridine (**3**), and the isolation of different isomeric forms. The complexes were synthesized rapidly in an Anton Paar Monowave 50 synthesis reactor in 5 minutes at 323 K and crystallized upon cooling the mother liquor.
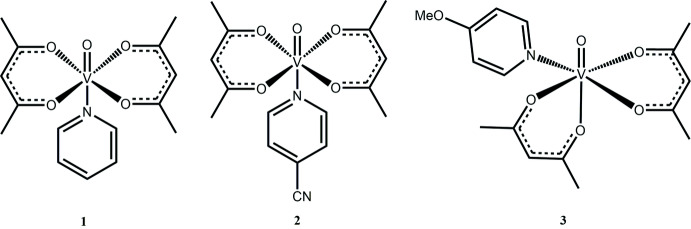


## Structural commentary   

**Figure 1:**
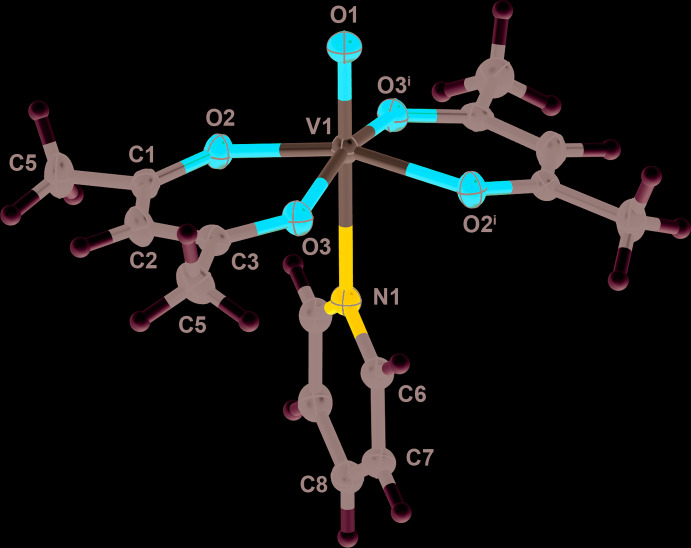
A view of compound **1**, showing the atom labeling. Displacement ellipsoids are at the 50% probability level and H atoms have been omitted for clarity. [Symmetry code (i) −*x* + 1, *y*, −*z* + 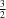].

Figs. 1 ▸–3 ▸ ▸ illustrate the mol­ecular structures of compounds **1**–**3**. Compounds **1** and **2** crystallize in the monoclinic space group *C*2/*c.* In both complexes, a twofold axis runs along the O—V—N bonding axis, leading to an asymmetric unit that consists of half of the mol­ecular structure. Upon symmetry expansion, both **1** and **2** adopt distorted octa­hedral geometries around the vanadium metal center with the oxo and pyridyl ligands *trans* to one another. Each acetyl­acetonate ligand chelates the vanadium center through two oxygen atoms to form a five-membered ring. In **1** and **2**, the equatorial plane consisting of the vanadium center and four acetyl­acetonate oxygen atoms distorts away from the V=O double bond. In **1**, the O_oxo_—V—O_acac_ bond angles are 98.05 (3)° and 99.84 (3)° and in **2** are 98.42 (4)° and 98.91 (3)°.

Compound **3** exists as a different isomeric form, with the oxo and 4-meth­oxy­pyridine ligand being *cis* to one another. This removes the twofold symmetry seen in compounds **1** and **2** and compound **3** crystallizes in the space group *P*2_1_/*n*. Similarly to **1** and **2**, compound **3** adopts a distorted octa­hedral geometry upon chelation by two bidentate acetyl­acetonate ligands.

The V—O and V=O bond lengths for **1**–**3** are are similar to those observed in related complexes (Singh *et al.*, 2014 ▸; Abakumova *et al.* 2012 ▸; Meicheng *et al.*, 1983 ▸; Silva *et al.*, 2013 ▸; Kadirova *et al.*, 2009 ▸). Most notable are variances in the V—N bond lengths in the complexes. In **1** and **2**, the V—N bond lengths are of similar nature at 2.3861 (16) and 2.4022 (15) Å, respectively. However in **3**, the V—N bond length is much shorter at 2.1140 (12) Å, likely from a combination of the *cis*-isomeric structure in **3** and the electron-donating meth­oxy group of the 4-meth­oxy­pyridine ligand.

## Supra­molecular features   

**Figure 4:**
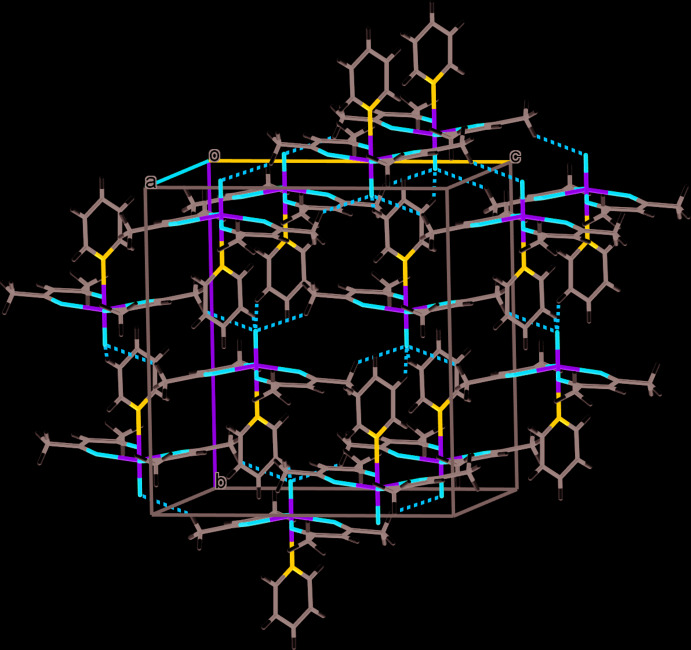
Crystal packing diagram of compound **1** with non-covalent inter­actions shown with dotted orange lines.

Several non-covalent inter­actions (Tables 1 ▸–3 ▸ ▸) exist in the supra­molecular structures of compounds **1**–**3**. Figs. 4 ▸–6 ▸ ▸ show the crystal packing diagrams for the compounds with the inter­actions shown as dashed orange lines. In **1**, these inter­actions are centered around the oxo ligand, with methyl groups of the acetyl­acetonate ligands forming CH_2_—H⋯O^ii^ inter­actions at a distance of 2.575 (9) Å between O1^ii^ and H5*A* and the aryl protons of the pyridine ligand forming Ar—H⋯O^iii^ inter­actions at a distance of 2.429 (9) Å between O1^iii^ and H7 [symmetry codes: (ii) −*x* + 1, −*y* + 1, −*z* + 1; (iii) *x* − 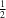, *y* − 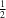, *z*]. Similar inter­actions exist in **2** with CH_2_—H⋯O^ii^ inter­actions at a distance of 2.591 (9) Å between the oxo ligand, O1^ii^, and H4*C* of the methyl group of the acetyl­acetonate and Ar—H⋯O^iii^ inter­actions at a distance of 2.588 (9) Å between the 4-cyano­pyridine proton H7 and O1^iii^ [symmetry codes: (ii) −*x* + 1, −*y* + 2, −*z* + 1; (iii) *x* − 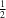, *y* − 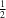, *z*]. Compound **2** also displays inter­actions between the methyl groups of the acetyl­acetonate and the π-bond within 4-cyano­pyridine at a distance of 2.682 (9) Å from the proton to the center of the π-bond.

Compared to **1** and **2**, compound **3** displays different types of non-covalent inter­actions. The methine proton, H2, of the acetyl­acetonate inter­acts with the oxo ligand oxygen, O1^i^ at a distance of 2.568 (8) Å and the aryl protons H12 and H14 inter­act with acetyl­acetonate oxygens atoms O2^ii^ and O4^iii^ at distances of 2.366 (8) Å and 2.569 (8) Å, respectively [symmetry codes: (i) −*x* + 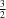, *y* + 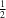, −*z* + 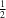; (ii) −*x* + 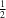, *y* − 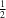, −*z* + 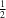; (iii) −*x* + 1, −*y* + 1, −*z* + 1]. Additionally, there are weaker inter­actions between the π-system of the acetyl­acetonate and 4-meth­oxy­pyridine, evident by a 3.196 (9) Å distance from C3 to C14^iii^.

## Synthesis and crystallization   

Bis(acetyl­acetonato)oxovanadium(IV) (VO(acac)_2_) and the N-donor ligands pyridine, 4-cyano­pyridine, and 4-meth­oxy­pyridine were purchased and used without further purification. To an Anton Paar Monowave synthesis reactor vial, a 1:1 molar ratio of VO(acac)_2_ and an N-donor ligand (0.75 mmol scale) was added and dissolved into 5 mL of di­chloro­methane. Once dissolved completely, each solution was reacted in an Anton Paar Monowave 50 synthesis reactor at 323 K for 5 min. Following all of the reactions, a slight precipitate was filtered and the resulting filtrate was allowed to slowly evaporate to produce single crystals suitable for X-ray diffraction studies. In addition to characterization by single crystal X-ray diffraction, each complex was characterized by FTIR spectroscopy. Compound **1** IR (neat) ν (cm^−1^): 3065(*w*), 2964(*w*), 1574(*m*), 1522(*s*), 1443(*m*), 1378(*s*) 1351(*s*), 1274(*m*), 1218(*w*), 1196(*w*), 1147(*w*), 1074(*w*), 1018(*m*), 964(*s*), 931(*m*), 890(*w*), 782(*w*), 763(*m*), 708(*m*), 676(*m*). Compound **2** IR (neat) ν(cm^−1^): 3084(*w*), 3034(*w*), 1557(*m*), 1540(*w*), 1522(*s*), 1411(*m*), 1375(*s*), 1277(*m*), 1211(*w*), 1190(*w*), 1018(*m*), 960(*s*), 929(*m*), 850(*m*), 789(*m*), 737(*w*), 679(*m*), 667(*m*). Compound **3** IR (neat) ν(cm^−1^): 3072(*w*), 3017(*w*), 1577(*m*), 1513(*s*), 1431(*m*), 1367(*s*), 1329(*m*), 1291(*m*), 1273(*m*), 1210(*m*), 1109(*w*), 1058(*w*), 1029(*m*), 948(*s*), 928(*m*), 836(*m*), 807(*m*), 780(*m*), 678(*w*), 658(*m*).

## Refinement   

Crystal data, data collection and structure refinement details are summarized in Table 4 ▸. Single crystals were examined under Infineum V8512 oil. The datum crystal was affixed to a MiTeGen loop and transferred to the cold nitro­gen stream of a Bruker APEXII diffractometer equipped with an Oxford Cryosystems 700 low-temperature apparatus. Unit-cell parameters were determined using reflections harvested from three sets of 12 0.5° ω scans scans. An optimal data-collection strategy was determined for an arbitrary hemisphere of data to 99.8% completeness to a resolution of 0.8 Å. (Bruker, 2015 ▸) Unit-cell parameters were refined using reflections harvested from the data collection with *I* ≥ 10σ(*I*). All data were corrected for Lorentz and polarization effects, and runs were scaled using *SADABS* (Krause *et al.*, 2015 ▸). The structures were solved using the Autostructure option within *APEX3*. This option employs an iterative application of the direct methods, Patterson synthesis, and dual-space routines of *SHELXT* (Sheldrick, 2015*a* ▸). The models were refined routinely (*SHELXL*; Sheldrick, 2015*b* ▸). Hydrogen atoms were placed at calculated geometries and allowed to ride on the position of the parent atom. Methyl H atoms were allowed to rotate but not to tip to best fit the experimental electron density. Hydrogen displacement parameters were set to 1.5*U*_eq_(C) for methyl and 1.2*U*_eq_(C) for all other hydrogen atoms.

## Supplementary Material

Crystal structure: contains datablock(s) compound1, compound2, compound3, global. DOI: 10.1107/S2056989020006246/zl2781sup1.cif

Structure factors: contains datablock(s) compound1. DOI: 10.1107/S2056989020006246/zl2781compound1sup2.hkl

Structure factors: contains datablock(s) compound2. DOI: 10.1107/S2056989020006246/zl2781compound2sup3.hkl

Structure factors: contains datablock(s) compound3. DOI: 10.1107/S2056989020006246/zl2781compound3sup4.hkl

CCDC references: 2002530, 2002529, 2002528

Additional supporting information:  crystallographic information; 3D view; checkCIF report

## Figures and Tables

**Figure 2:**
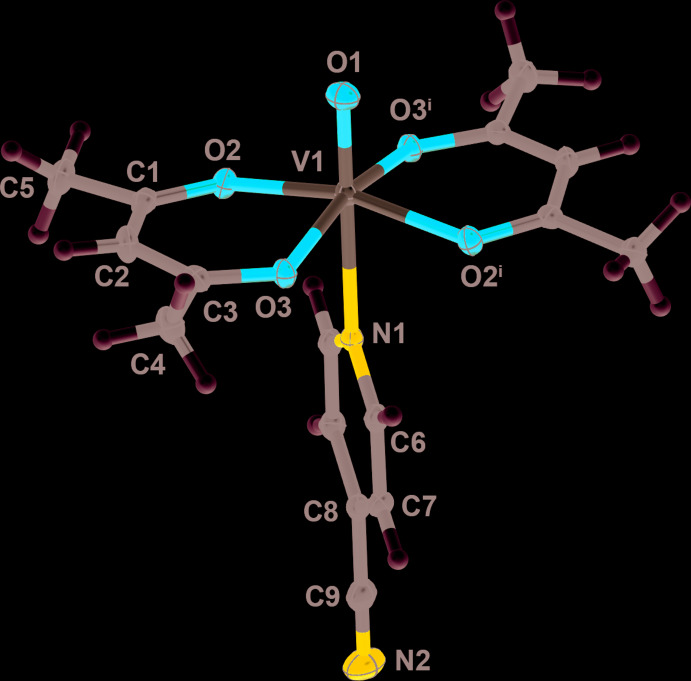
A view of compound **2**, showing the atom labeling. Displacement ellipsoids are at the 50% probability level and H atoms have been omitted for clarity. [Symmetry code (i) −*x* + 1, *y*, −*z* + 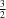].

**Figure 3:**
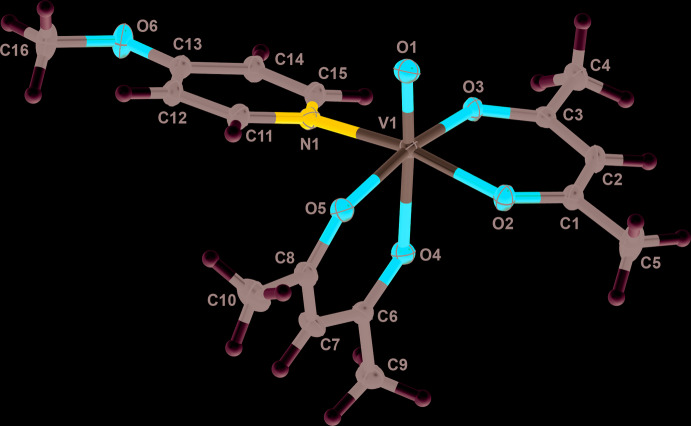
A view of compound **3**, showing the atom labeling. Displacement ellipsoids are at the 50% probability level and H atoms have been omitted for clarity.

**Figure 5:**
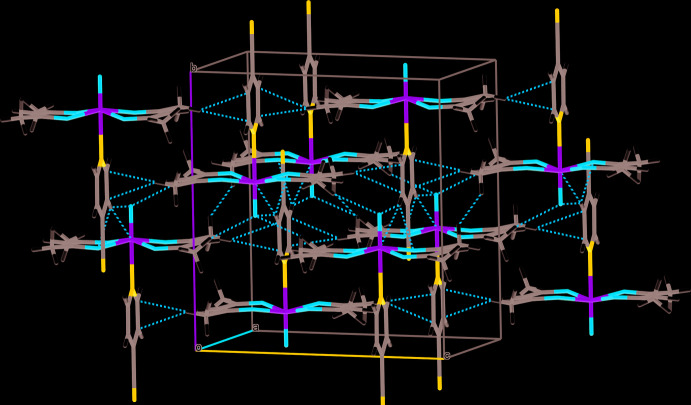
Crystal packing diagram of compound **2** with non-covalent inter­actions shown with dotted orange lines.

**Figure 6:**
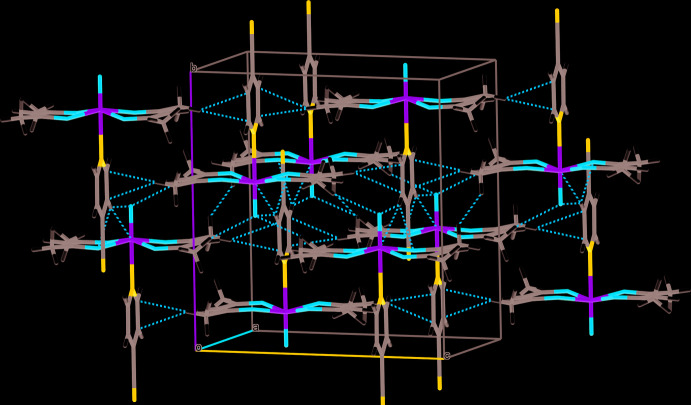
Crystal packing diagram of compound **3** with non-covalent inter­actions shown with dotted orange lines.

**Table 1:** Hydrogen-bond geometry (Å, °) for compound **1** [Chem scheme1]

*D*—H⋯*A*	*D*—H	H⋯*A*	*D*⋯*A*	*D*—H⋯*A*
C5—H5*A*⋯O1^ii^	0.98	2.58	3.2589 (18)	127
C7—H7⋯O1^iii^	0.95	2.43	3.2487 (17)	144

Symmetry codes: (ii) 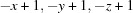; (iii) 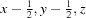.

**Table 2:** Hydrogen-bond geometry (Å, °) for compound **2** [Chem scheme1]

*D*—H⋯*A*	*D*—H	H⋯*A*	*D*⋯*A*	*D*—H⋯*A*
C4—H4*C*⋯O1^ii^	0.98	2.47	3.4249 (16)	164
C7—H7⋯O1^iii^	0.95	2.59	3.4673 (14)	154

Symmetry codes: (ii) 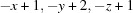; (iii) 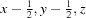.

**Table 3:** Hydrogen-bond geometry (Å, °) for compound **3** [Chem scheme1]

*D*—H⋯*A*	*D*—H	H⋯*A*	*D*⋯*A*	*D*—H⋯*A*
C2—H2⋯O1^i^	0.95	2.57	3.488 (2)	163
C12—H12⋯O2^ii^	0.95	2.37	3.2567 (18)	156
C14—H14⋯O4^iii^	0.95	2.57	3.2462 (18)	129

Symmetry codes: (i) 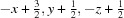; (ii) 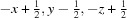; (iii) 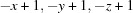.

**Table 4:** Experimental details

	**1**	**2**	**3**
Crystal data			
Chemical formula	[V(C_5_H_7_O_2_)_2_O(C_5_H_5_N)]	[V(C_5_H_7_O_2_)_2_O(C_6_H_4_N_2_)]	[V(C_5_H_7_O_2_)_2_O(C_6_H_7_NO)]
*M*_r_	344.25	369.26	374.28
Crystal system, space group	Monoclinic, *C*2/*c*	Monoclinic, *C*2/*c*	Monoclinic, *P*2_1_/*n*
Temperature (K)	120	120	120
*a*, *b*, *c* (Å)	7.8820 (5), 15.2092 (11), 13.9871 (9)	9.1930 (9), 13.5080 (9), 13.3651 (9)	9.6619 (15), 11.9922 (19), 15.344 (2)
β (°)	103.367 (2)	99.030 (3)	94.651 (2)
*V* (Å^3^)	1631.33 (19)	1639.1 (2)	1772.0 (5)
*Z*	4	4	4
Radiation type	Mo *K*α	Mo *K*α	Mo *K*α
μ (mm^−1^)	0.63	0.63	0.59
Crystal size (mm)	0.23 × 0.14 × 0.11	0.15 × 0.11 × 0.07	0.32 × 0.19 × 0.11

Data collection			
Diffractometer	Bruker APEXII	Bruker APEXII	Bruker Kappa X8-APEXII
Absorption correction	Numerical (*SADABS*; Krause *et al.*, 2015 ▸)	Numerical (*SADABS*; Krause *et al.*, 2015 ▸)	Numerical (*SADABS*; Krause *et al.*, 2015 ▸)
*T*_min_, *T*_max_	0.912, 0.966	0.927, 0.980	0.862, 0.983
No. of measured, independent and observed [*I* > 2σ(*I*)] reflections	17837, 2037, 1882	21763, 2038, 1832	25688, 4413, 3730
*R*_int_	0.025	0.032	0.033
(sin θ/λ)_max_ (Å^−1^)	0.667	0.667	0.668

Refinement			
*R*[*F* ^2^ > 2σ(*F* ^2^)], *wR*(*F* ^2^), *S*	0.027, 0.073, 1.06	0.027, 0.071, 1.08	0.030, 0.081, 1.05
No. of reflections	2037	2038	4413
No. of parameters	104	114	222
H-atom treatment	H-atom parameters constrained	H-atom parameters constrained	H-atom parameters constrained
Δρ_max_, Δρ_min_ (e Å^−3^)	0.31, −0.26	0.41, −0.40	0.33, −0.36

Computer programs: *APEX3* and *SAINT* (Bruker, 2015 ▸), *SHELXT2018/2* (Sheldrick, 2015*a* ▸), *SHELXL2018/3* (Sheldrick, 2015*b* ▸), *XP* (Bruker, 2015 ▸), *Mercury* (Macrae *et al.*, 2020 ▸) and *publCIF* (Westrip, 2010 ▸).

## supplementary crystallographic information

### Bis(acetylacetonato-κ2O,O')oxido(pyridine-κN)vanadium(IV) (compound1) Crystal data

**Table:**

[V(C_5_H_7_O_2_)_2_O(C_5_H_5_N)]	*F*(000) = 716
*M**_r_* = 344.25	*D*_x_ = 1.402 Mg m^−^^3^
Monoclinic, *C*2/*c*	Mo *K*α radiation, λ = 0.71073 Å
*a* = 7.8820 (5) Å	Cell parameters from 8119 reflections
*b* = 15.2092 (11) Å	θ = 2.7–28.3°
*c* = 13.9871 (9) Å	µ = 0.63 mm^−^^1^
β = 103.367 (2)°	*T* = 120 K
*V* = 1631.33 (19) Å^3^	Tablet, blue
*Z* = 4	0.23 × 0.14 × 0.11 mm

### Bis(acetylacetonato-κ2O,O')oxido(pyridine-κN)vanadium(IV) (compound1) Data collection

**Table:**

Bruker APEXII diffractometer	2037 independent reflections
Radiation source: fine-focus sealed tube	1882 reflections with *I* > 2σ(*I*)
Detector resolution: 8.33 pixels mm^-1^	*R*_int_ = 0.025
combination of ω and φ–scans	θ_max_ = 28.3°, θ_min_ = 2.7°
Absorption correction: numerical (SADABS; Krause *et al.*, 2015)	*h* = −10→10
*T*_min_ = 0.912, *T*_max_ = 0.966	*k* = −20→20
17837 measured reflections	*l* = −18→18

### Bis(acetylacetonato-κ2O,O')oxido(pyridine-κN)vanadium(IV) (compound1) Refinement

**Table:**

Refinement on *F*^2^	Primary atom site location: dual
Least-squares matrix: full	Secondary atom site location: difference Fourier map
*R*[*F*^2^ > 2σ(*F*^2^)] = 0.027	Hydrogen site location: inferred from neighbouring sites
*wR*(*F*^2^) = 0.073	H-atom parameters constrained
*S* = 1.06	*w* = 1/[σ^2^(*F*_o_^2^) + (0.0362*P*)^2^ + 1.4639*P*] where *P* = (*F*_o_^2^ + 2*F*_c_^2^)/3
2037 reflections	(Δ/σ)_max_ < 0.001
104 parameters	Δρ_max_ = 0.31 e Å^−^^3^
0 restraints	Δρ_min_ = −0.26 e Å^−^^3^

### Bis(acetylacetonato-κ2O,O')oxido(pyridine-κN)vanadium(IV) (compound1) Special details

**Table:**

Geometry. All esds (except the esd in the dihedral angle between two l.s. planes) are estimated using the full covariance matrix. The cell esds are taken into account individually in the estimation of esds in distances, angles and torsion angles; correlations between esds in cell parameters are only used when they are defined by crystal symmetry. An approximate (isotropic) treatment of cell esds is used for estimating esds involving l.s. planes.

### Bis(acetylacetonato-κ2O,O')oxido(pyridine-κN)vanadium(IV) (compound1) Fractional atomic coordinates and isotropic or equivalent isotropic displacement parameters (Å^2^)

**Table:**

	*x*	*y*	*z*	*U*_iso_*/*U*_eq_	
V1	0.500000	0.41643 (2)	0.750000	0.01694 (10)	
O1	0.500000	0.52179 (9)	0.750000	0.0239 (3)	
O2	0.54371 (12)	0.39403 (6)	0.61740 (7)	0.0211 (2)	
O3	0.24437 (11)	0.39799 (6)	0.69551 (7)	0.0212 (2)	
N1	0.500000	0.25955 (10)	0.750000	0.0193 (3)	
C1	0.43127 (17)	0.37920 (9)	0.53797 (9)	0.0209 (3)	
C2	0.25102 (18)	0.37547 (10)	0.52875 (10)	0.0264 (3)	
H2	0.179805	0.365061	0.465064	0.032*	
C3	0.16766 (17)	0.38580 (8)	0.60560 (10)	0.0211 (3)	
C4	−0.02841 (18)	0.38382 (11)	0.58352 (12)	0.0312 (3)	
H4A	−0.066433	0.356674	0.638591	0.047*	
H4B	−0.072855	0.349511	0.523659	0.047*	
H4C	−0.073646	0.443987	0.573924	0.047*	
C5	0.5033 (2)	0.36549 (12)	0.44857 (11)	0.0322 (3)	
H5A	0.542558	0.421933	0.427631	0.048*	
H5B	0.412268	0.340944	0.395398	0.048*	
H5C	0.601990	0.324655	0.464361	0.048*	
C6	0.37706 (16)	0.21376 (9)	0.78046 (9)	0.0224 (3)	
H6	0.289206	0.245242	0.802491	0.027*	
C7	0.37197 (19)	0.12280 (10)	0.78135 (10)	0.0274 (3)	
H7	0.282169	0.092712	0.803098	0.033*	
C8	0.500000	0.07661 (13)	0.750000	0.0293 (4)	
H8	0.500002	0.014145	0.750000	0.035*	

### Bis(acetylacetonato-κ2O,O')oxido(pyridine-κN)vanadium(IV) (compound1) Atomic displacement parameters (Å^2^)

**Table:**

	*U*^11^	*U*^22^	*U*^33^	*U*^12^	*U*^13^	*U*^23^
V1	0.01327 (15)	0.02129 (16)	0.01593 (15)	0.000	0.00271 (10)	0.000
O1	0.0223 (6)	0.0235 (7)	0.0260 (7)	0.000	0.0059 (5)	0.000
O2	0.0176 (4)	0.0282 (5)	0.0176 (4)	−0.0010 (3)	0.0040 (3)	0.0010 (3)
O3	0.0142 (4)	0.0279 (5)	0.0207 (4)	0.0012 (3)	0.0021 (3)	−0.0002 (4)
N1	0.0155 (7)	0.0221 (7)	0.0198 (7)	0.000	0.0033 (5)	0.000
C1	0.0230 (6)	0.0206 (6)	0.0185 (6)	0.0018 (5)	0.0037 (5)	0.0021 (5)
C2	0.0206 (6)	0.0354 (8)	0.0203 (6)	0.0013 (5)	−0.0014 (5)	−0.0029 (5)
C3	0.0170 (6)	0.0195 (6)	0.0249 (6)	0.0011 (4)	0.0004 (5)	0.0001 (5)
C4	0.0163 (6)	0.0408 (8)	0.0333 (8)	0.0005 (6)	−0.0004 (6)	−0.0075 (6)
C5	0.0312 (8)	0.0463 (9)	0.0195 (6)	0.0008 (7)	0.0069 (6)	−0.0013 (6)
C6	0.0170 (6)	0.0276 (7)	0.0227 (6)	−0.0019 (5)	0.0047 (5)	−0.0003 (5)
C7	0.0286 (7)	0.0284 (7)	0.0244 (7)	−0.0090 (5)	0.0043 (5)	0.0005 (5)
C8	0.0403 (12)	0.0217 (9)	0.0228 (9)	0.000	0.0013 (8)	0.000

### Bis(acetylacetonato-κ2O,O')oxido(pyridine-κN)vanadium(IV) (compound1) Geometric parameters (Å, º)

**Table:**

V1—O1	1.6024 (14)	C2—H2	0.9500
V1—O2	1.9925 (9)	C3—C4	1.5047 (18)
V1—O2^i^	1.9925 (9)	C4—H4A	0.9800
V1—O3^i^	2.0023 (9)	C4—H4B	0.9800
V1—O3	2.0023 (9)	C4—H4C	0.9800
V1—N1	2.3861 (16)	C5—H5A	0.9800
O2—C1	1.2710 (16)	C5—H5B	0.9800
O3—C3	1.2766 (16)	C5—H5C	0.9800
N1—C6	1.3402 (15)	C6—C7	1.384 (2)
N1—C6^i^	1.3402 (15)	C6—H6	0.9500
C1—C2	1.3977 (19)	C7—C8	1.3816 (18)
C1—C5	1.5025 (19)	C7—H7	0.9500
C2—C3	1.392 (2)	C8—H8	0.9500
			
O1—V1—O2	99.84 (3)	C1—C2—H2	117.4
O1—V1—O2^i^	99.85 (3)	O3—C3—C2	125.19 (12)
O2—V1—O2^i^	160.31 (6)	O3—C3—C4	115.80 (12)
O1—V1—O3^i^	98.05 (3)	C2—C3—C4	119.01 (12)
O2—V1—O3^i^	87.43 (4)	C3—C4—H4A	109.5
O2^i^—V1—O3^i^	89.83 (4)	C3—C4—H4B	109.5
O1—V1—O3	98.05 (3)	H4A—C4—H4B	109.5
O2—V1—O3	89.83 (4)	C3—C4—H4C	109.5
O2^i^—V1—O3	87.42 (4)	H4A—C4—H4C	109.5
O3^i^—V1—O3	163.90 (6)	H4B—C4—H4C	109.5
O1—V1—N1	180.0	C1—C5—H5A	109.5
O2—V1—N1	80.16 (3)	C1—C5—H5B	109.5
O2^i^—V1—N1	80.15 (3)	H5A—C5—H5B	109.5
O3^i^—V1—N1	81.95 (3)	C1—C5—H5C	109.5
O3—V1—N1	81.95 (3)	H5A—C5—H5C	109.5
C1—O2—V1	127.47 (9)	H5B—C5—H5C	109.5
C3—O3—V1	126.95 (9)	N1—C6—C7	123.15 (13)
C6—N1—C6^i^	117.39 (16)	N1—C6—H6	118.4
C6—N1—V1	121.31 (8)	C7—C6—H6	118.4
C6^i^—N1—V1	121.30 (8)	C8—C7—C6	118.72 (14)
O2—C1—C2	125.19 (12)	C8—C7—H7	120.6
O2—C1—C5	115.52 (12)	C6—C7—H7	120.6
C2—C1—C5	119.28 (12)	C7—C8—C7^i^	118.87 (19)
C3—C2—C1	125.12 (13)	C7—C8—H8	120.6
C3—C2—H2	117.4	C7^i^—C8—H8	120.6
			
V1—O2—C1—C2	−0.2 (2)	C1—C2—C3—O3	−1.8 (2)
V1—O2—C1—C5	179.40 (9)	C1—C2—C3—C4	177.47 (13)
O2—C1—C2—C3	−1.3 (2)	C6^i^—N1—C6—C7	−0.21 (9)
C5—C1—C2—C3	179.11 (14)	V1—N1—C6—C7	179.79 (9)
V1—O3—C3—C2	5.89 (19)	N1—C6—C7—C8	0.41 (18)
V1—O3—C3—C4	−173.40 (9)	C6—C7—C8—C7^i^	−0.19 (9)

Symmetry code: (i) −*x*+1, *y*, −*z*+3/2.

### Bis(acetylacetonato-κ2O,O')oxido(pyridine-κN)vanadium(IV) (compound1) Hydrogen-bond geometry (Å, º)

**Table:**

*D*—H···*A*	*D*—H	H···*A*	*D*···*A*	*D*—H···*A*
C5—H5*A*···O1^ii^	0.98	2.58	3.2589 (18)	127
C7—H7···O1^iii^	0.95	2.43	3.2487 (17)	144

Symmetry codes: (ii) −*x*+1, −*y*+1, −*z*+1; (iii) *x*−1/2, *y*−1/2, *z*.

### Bis(acetylacetonato-κ2O,O')oxido(pyridine-4-carbonitrile-κN)vanadium(IV) (compound2) Crystal data

**Table:**

[V(C_5_H_7_O_2_)_2_O(C_6_H_4_N_2_)]	*F*(000) = 764
*M**_r_* = 369.26	*D*_x_ = 1.496 Mg m^−^^3^
Monoclinic, *C*2/*c*	Mo *K*α radiation, λ = 0.71073 Å
*a* = 9.1930 (9) Å	Cell parameters from 7973 reflections
*b* = 13.5080 (9) Å	θ = 2.7–28.1°
*c* = 13.3651 (9) Å	µ = 0.63 mm^−^^1^
β = 99.030 (3)°	*T* = 120 K
*V* = 1639.1 (2) Å^3^	Block, green
*Z* = 4	0.15 × 0.11 × 0.07 mm

### Bis(acetylacetonato-κ2O,O')oxido(pyridine-4-carbonitrile-κN)vanadium(IV) (compound2) Data collection

**Table:**

Bruker APEXII diffractometer	2038 independent reflections
Radiation source: fine-focus sealed tube	1832 reflections with *I* > 2σ(*I*)
Detector resolution: 8.33 pixels mm^-1^	*R*_int_ = 0.032
combination of ω and φ–scans	θ_max_ = 28.3°, θ_min_ = 2.7°
Absorption correction: numerical (SADABS; Krause *et al.*, 2015)	*h* = −12→12
*T*_min_ = 0.927, *T*_max_ = 0.980	*k* = −18→18
21763 measured reflections	*l* = −17→17

### Bis(acetylacetonato-κ2O,O')oxido(pyridine-4-carbonitrile-κN)vanadium(IV) (compound2) Refinement

**Table:**

Refinement on *F*^2^	Primary atom site location: dual
Least-squares matrix: full	Secondary atom site location: difference Fourier map
*R*[*F*^2^ > 2σ(*F*^2^)] = 0.027	Hydrogen site location: inferred from neighbouring sites
*wR*(*F*^2^) = 0.071	H-atom parameters constrained
*S* = 1.08	*w* = 1/[σ^2^(*F*_o_^2^) + (0.0324*P*)^2^ + 1.6979*P*] where *P* = (*F*_o_^2^ + 2*F*_c_^2^)/3
2038 reflections	(Δ/σ)_max_ = 0.001
114 parameters	Δρ_max_ = 0.41 e Å^−^^3^
0 restraints	Δρ_min_ = −0.40 e Å^−^^3^

### Bis(acetylacetonato-κ2O,O')oxido(pyridine-4-carbonitrile-κN)vanadium(IV) (compound2) Special details

**Table:**

Geometry. All esds (except the esd in the dihedral angle between two l.s. planes) are estimated using the full covariance matrix. The cell esds are taken into account individually in the estimation of esds in distances, angles and torsion angles; correlations between esds in cell parameters are only used when they are defined by crystal symmetry. An approximate (isotropic) treatment of cell esds is used for estimating esds involving l.s. planes.

### Bis(acetylacetonato-κ2O,O')oxido(pyridine-4-carbonitrile-κN)vanadium(IV) (compound2) Fractional atomic coordinates and isotropic or equivalent isotropic displacement parameters (Å^2^)

**Table:**

	*x*	*y*	*z*	*U*_iso_*/*U*_eq_	
V1	0.500000	0.89112 (2)	0.750000	0.01271 (10)	
O1	0.500000	1.00987 (10)	0.750000	0.0210 (3)	
O2	0.60596 (10)	0.86813 (7)	0.63185 (7)	0.0166 (2)	
O3	0.31046 (10)	0.86962 (7)	0.65896 (7)	0.0151 (2)	
N1	0.500000	0.71328 (11)	0.750000	0.0130 (3)	
N2	0.500000	0.31564 (14)	0.750000	0.0318 (4)	
C1	0.54997 (15)	0.87153 (9)	0.53874 (10)	0.0157 (3)	
C2	0.39870 (16)	0.87878 (10)	0.50203 (10)	0.0195 (3)	
H2	0.369478	0.886023	0.431015	0.023*	
C3	0.28850 (15)	0.87607 (9)	0.56257 (10)	0.0155 (3)	
C4	0.65451 (16)	0.86478 (11)	0.46315 (11)	0.0214 (3)	
H4A	0.653674	0.797228	0.436328	0.032*	
H4B	0.754227	0.881506	0.496340	0.032*	
H4C	0.623837	0.911208	0.407492	0.032*	
C5	0.12947 (16)	0.88186 (11)	0.51467 (11)	0.0223 (3)	
H5A	0.121409	0.870964	0.441516	0.033*	
H5B	0.090305	0.947415	0.527156	0.033*	
H5C	0.073029	0.830993	0.544114	0.033*	
C6	0.37746 (14)	0.66216 (10)	0.75924 (9)	0.0148 (3)	
H6	0.290548	0.697948	0.765924	0.018*	
C7	0.37194 (14)	0.55958 (10)	0.75947 (10)	0.0164 (3)	
H7	0.283303	0.525726	0.765917	0.020*	
C8	0.500000	0.50744 (14)	0.750000	0.0161 (4)	
C9	0.500000	0.40014 (15)	0.750000	0.0222 (4)	

### Bis(acetylacetonato-κ2O,O')oxido(pyridine-4-carbonitrile-κN)vanadium(IV) (compound2) Atomic displacement parameters (Å^2^)

**Table:**

	*U*^11^	*U*^22^	*U*^33^	*U*^12^	*U*^13^	*U*^23^
V1	0.01153 (15)	0.01478 (16)	0.01141 (15)	0.000	0.00052 (11)	0.000
O1	0.0213 (7)	0.0169 (7)	0.0235 (7)	0.000	−0.0005 (6)	0.000
O2	0.0149 (4)	0.0214 (5)	0.0137 (4)	0.0006 (3)	0.0032 (4)	0.0027 (3)
O3	0.0127 (4)	0.0194 (5)	0.0126 (4)	0.0007 (3)	0.0002 (3)	0.0002 (3)
N1	0.0133 (7)	0.0150 (7)	0.0108 (7)	0.000	0.0026 (5)	0.000
N2	0.0394 (11)	0.0174 (9)	0.0429 (12)	0.000	0.0196 (9)	0.000
C1	0.0198 (6)	0.0124 (6)	0.0158 (6)	0.0018 (5)	0.0055 (5)	0.0022 (4)
C2	0.0215 (7)	0.0261 (7)	0.0108 (6)	0.0043 (5)	0.0016 (5)	0.0008 (5)
C3	0.0170 (6)	0.0136 (6)	0.0150 (6)	0.0023 (4)	−0.0009 (5)	−0.0013 (5)
C4	0.0248 (7)	0.0232 (7)	0.0179 (7)	0.0058 (5)	0.0092 (6)	0.0039 (5)
C5	0.0171 (6)	0.0310 (8)	0.0168 (7)	0.0039 (5)	−0.0031 (5)	−0.0014 (6)
C6	0.0134 (6)	0.0181 (6)	0.0135 (6)	0.0005 (5)	0.0035 (5)	0.0003 (5)
C7	0.0162 (6)	0.0186 (6)	0.0151 (6)	−0.0032 (5)	0.0044 (5)	0.0001 (5)
C8	0.0219 (9)	0.0148 (9)	0.0121 (8)	0.000	0.0042 (7)	0.000
C9	0.0257 (10)	0.0212 (10)	0.0218 (10)	0.000	0.0107 (8)	0.000

### Bis(acetylacetonato-κ2O,O')oxido(pyridine-4-carbonitrile-κN)vanadium(IV) (compound2) Geometric parameters (Å, º)

**Table:**

V1—O1	1.6040 (14)	C2—H2	0.9500
V1—O3^i^	1.9838 (9)	C3—C5	1.5031 (18)
V1—O3	1.9838 (9)	C4—H4A	0.9800
V1—O2^i^	2.0055 (9)	C4—H4B	0.9800
V1—O2	2.0055 (9)	C4—H4C	0.9800
V1—N1	2.4022 (15)	C5—H5A	0.9800
O2—C1	1.2706 (17)	C5—H5B	0.9800
O3—C3	1.2752 (17)	C5—H5C	0.9800
N1—C6	1.3435 (15)	C6—C7	1.3866 (19)
N1—C6^i^	1.3435 (15)	C6—H6	0.9500
N2—C9	1.141 (3)	C7—C8	1.3946 (16)
C1—C2	1.4035 (19)	C7—H7	0.9500
C1—C4	1.5024 (18)	C8—C9	1.449 (3)
C2—C3	1.3928 (19)		
			
O1—V1—O3^i^	98.42 (3)	O3—C3—C2	125.06 (13)
O1—V1—O3	98.42 (3)	O3—C3—C5	115.03 (12)
O3^i^—V1—O3	163.17 (6)	C2—C3—C5	119.91 (12)
O1—V1—O2^i^	98.91 (3)	C1—C4—H4A	109.5
O3^i^—V1—O2^i^	89.01 (4)	C1—C4—H4B	109.5
O3—V1—O2^i^	88.39 (4)	H4A—C4—H4B	109.5
O1—V1—O2	98.91 (3)	C1—C4—H4C	109.5
O3^i^—V1—O2	88.39 (4)	H4A—C4—H4C	109.5
O3—V1—O2	89.01 (4)	H4B—C4—H4C	109.5
O2^i^—V1—O2	162.19 (6)	C3—C5—H5A	109.5
O1—V1—N1	180.0	C3—C5—H5B	109.5
O3^i^—V1—N1	81.58 (3)	H5A—C5—H5B	109.5
O3—V1—N1	81.58 (3)	C3—C5—H5C	109.5
O2^i^—V1—N1	81.09 (3)	H5A—C5—H5C	109.5
O2—V1—N1	81.09 (3)	H5B—C5—H5C	109.5
C1—O2—V1	126.40 (9)	N1—C6—C7	123.05 (12)
C3—O3—V1	126.45 (9)	N1—C6—H6	118.5
C6—N1—C6^i^	118.13 (15)	C7—C6—H6	118.5
C6—N1—V1	120.93 (8)	C6—C7—C8	118.22 (12)
C6^i^—N1—V1	120.93 (8)	C6—C7—H7	120.9
O2—C1—C2	124.87 (12)	C8—C7—H7	120.9
O2—C1—C4	116.92 (12)	C7^i^—C8—C7	119.33 (17)
C2—C1—C4	118.19 (12)	C7^i^—C8—C9	120.33 (8)
C3—C2—C1	124.49 (13)	C7—C8—C9	120.33 (8)
C3—C2—H2	117.8	N2—C9—C8	180.0
C1—C2—H2	117.8		
			
V1—O2—C1—C2	−9.23 (18)	C1—C2—C3—C5	−178.42 (13)
V1—O2—C1—C4	172.30 (9)	C6^i^—N1—C6—C7	−0.12 (9)
O2—C1—C2—C3	−4.8 (2)	V1—N1—C6—C7	179.88 (9)
C4—C1—C2—C3	173.61 (13)	N1—C6—C7—C8	0.22 (17)
V1—O3—C3—C2	14.38 (18)	C6—C7—C8—C7^i^	−0.11 (8)
V1—O3—C3—C5	−165.06 (9)	C6—C7—C8—C9	179.89 (8)
C1—C2—C3—O3	2.2 (2)		

Symmetry code: (i) −*x*+1, *y*, −*z*+3/2.

### Bis(acetylacetonato-κ2O,O')oxido(pyridine-4-carbonitrile-κN)vanadium(IV) (compound2) Hydrogen-bond geometry (Å, º)

**Table:**

*D*—H···*A*	*D*—H	H···*A*	*D*···*A*	*D*—H···*A*
C4—H4*C*···O1^ii^	0.98	2.47	3.4249 (16)	164
C7—H7···O1^iii^	0.95	2.59	3.4673 (14)	154

Symmetry codes: (ii) −*x*+1, −*y*+2, −*z*+1; (iii) *x*−1/2, *y*−1/2, *z*.

### Bis(acetylacetonato-κ2O,O')(4-methoxypyridine-κN)oxidovanadium(IV) (compound3) Crystal data

**Table:**

[V(C_5_H_7_O_2_)_2_O(C_6_H_7_NO)]	*F*(000) = 780
*M**_r_* = 374.28	*D*_x_ = 1.403 Mg m^−^^3^
Monoclinic, *P*2_1_/*n*	Mo *K*α radiation, λ = 0.71073 Å
*a* = 9.6619 (15) Å	Cell parameters from 9171 reflections
*b* = 11.9922 (19) Å	θ = 2.4–28.3°
*c* = 15.344 (2) Å	µ = 0.59 mm^−^^1^
β = 94.651 (2)°	*T* = 120 K
*V* = 1772.0 (5) Å^3^	Block, blue
*Z* = 4	0.32 × 0.19 × 0.11 mm

### Bis(acetylacetonato-κ2O,O')(4-methoxypyridine-κN)oxidovanadium(IV) (compound3) Data collection

**Table:**

Bruker Kappa X8-APEXII diffractometer	4413 independent reflections
Radiation source: fine-focus sealed tube	3730 reflections with *I* > 2σ(*I*)
Graphite monochromator	*R*_int_ = 0.033
Detector resolution: 8.33 pixels mm^-1^	θ_max_ = 28.4°, θ_min_ = 2.2°
combination of ω and φ–scans	*h* = −12→12
Absorption correction: numerical (SADABS; Krause *et al.*, 2015)	*k* = −16→16
*T*_min_ = 0.862, *T*_max_ = 0.983	*l* = −20→20
25688 measured reflections	

### Bis(acetylacetonato-κ2O,O')(4-methoxypyridine-κN)oxidovanadium(IV) (compound3) Refinement

**Table:**

Refinement on *F*^2^	Primary atom site location: dual
Least-squares matrix: full	Secondary atom site location: difference Fourier map
*R*[*F*^2^ > 2σ(*F*^2^)] = 0.030	Hydrogen site location: inferred from neighbouring sites
*wR*(*F*^2^) = 0.081	H-atom parameters constrained
*S* = 1.05	*w* = 1/[σ^2^(*F*_o_^2^) + (0.0348*P*)^2^ + 1.1201*P*] where *P* = (*F*_o_^2^ + 2*F*_c_^2^)/3
4413 reflections	(Δ/σ)_max_ < 0.001
222 parameters	Δρ_max_ = 0.33 e Å^−^^3^
0 restraints	Δρ_min_ = −0.36 e Å^−^^3^

### Bis(acetylacetonato-κ2O,O')(4-methoxypyridine-κN)oxidovanadium(IV) (compound3) Special details

**Table:**

Geometry. All esds (except the esd in the dihedral angle between two l.s. planes) are estimated using the full covariance matrix. The cell esds are taken into account individually in the estimation of esds in distances, angles and torsion angles; correlations between esds in cell parameters are only used when they are defined by crystal symmetry. An approximate (isotropic) treatment of cell esds is used for estimating esds involving l.s. planes.

### Bis(acetylacetonato-κ2O,O')(4-methoxypyridine-κN)oxidovanadium(IV) (compound3) Fractional atomic coordinates and isotropic or equivalent isotropic displacement parameters (Å^2^)

**Table:**

	*x*	*y*	*z*	*U*_iso_*/*U*_eq_	
V1	0.46984 (2)	0.46573 (2)	0.27536 (2)	0.01335 (7)	
O1	0.53209 (11)	0.36808 (9)	0.21879 (7)	0.0206 (2)	
O2	0.52992 (10)	0.59883 (9)	0.20819 (6)	0.0187 (2)	
O3	0.63443 (10)	0.49106 (8)	0.36048 (7)	0.0167 (2)	
O4	0.37408 (11)	0.59132 (8)	0.35341 (7)	0.0175 (2)	
O5	0.28270 (11)	0.46703 (8)	0.20760 (6)	0.0173 (2)	
O6	0.26958 (12)	0.12492 (9)	0.54929 (7)	0.0244 (2)	
N1	0.39173 (13)	0.35591 (10)	0.37014 (8)	0.0153 (2)	
C1	0.63492 (16)	0.66210 (12)	0.22605 (10)	0.0185 (3)	
C2	0.73408 (16)	0.64880 (13)	0.29670 (10)	0.0198 (3)	
H2	0.809349	0.699961	0.302343	0.024*	
C3	0.72947 (15)	0.56495 (12)	0.35957 (9)	0.0161 (3)	
C4	0.84276 (16)	0.55651 (13)	0.43262 (10)	0.0222 (3)	
H4A	0.801910	0.538371	0.487292	0.033*	
H4B	0.891915	0.627884	0.439042	0.033*	
H4C	0.908201	0.497767	0.419094	0.033*	
C5	0.6492 (2)	0.75837 (15)	0.16467 (12)	0.0334 (4)	
H5A	0.656443	0.730050	0.105296	0.050*	
H5B	0.732960	0.801013	0.183335	0.050*	
H5C	0.567632	0.806796	0.165362	0.050*	
C6	0.24885 (15)	0.62275 (12)	0.35059 (9)	0.0167 (3)	
C7	0.14287 (16)	0.58216 (14)	0.29076 (10)	0.0215 (3)	
H7	0.050317	0.605404	0.297396	0.026*	
C8	0.16543 (16)	0.51020 (13)	0.22263 (10)	0.0193 (3)	
C9	0.21222 (17)	0.71170 (13)	0.41458 (10)	0.0226 (3)	
H9A	0.242406	0.687897	0.474206	0.034*	
H9B	0.111497	0.723372	0.409565	0.034*	
H9C	0.258989	0.781507	0.401452	0.034*	
C10	0.04535 (18)	0.47785 (17)	0.15869 (12)	0.0320 (4)	
H10A	0.069407	0.492688	0.098957	0.048*	
H10B	−0.036755	0.521553	0.170540	0.048*	
H10C	0.025355	0.398275	0.165035	0.048*	
C11	0.28395 (15)	0.28757 (12)	0.34893 (9)	0.0177 (3)	
H11	0.238256	0.293682	0.291934	0.021*	
C12	0.23542 (15)	0.20886 (12)	0.40496 (10)	0.0184 (3)	
H12	0.157914	0.163143	0.387202	0.022*	
C13	0.30337 (15)	0.19861 (12)	0.48803 (9)	0.0171 (3)	
C14	0.41468 (15)	0.26970 (12)	0.51178 (9)	0.0178 (3)	
H14	0.462236	0.265216	0.568336	0.021*	
C15	0.45407 (15)	0.34604 (12)	0.45204 (9)	0.0174 (3)	
H15	0.529131	0.394613	0.469025	0.021*	
C16	0.15152 (19)	0.05480 (15)	0.52768 (11)	0.0289 (4)	
H16A	0.069247	0.101227	0.513676	0.043*	
H16B	0.136044	0.006690	0.577595	0.043*	
H16C	0.168604	0.008556	0.476993	0.043*	

### Bis(acetylacetonato-κ2O,O')(4-methoxypyridine-κN)oxidovanadium(IV) (compound3) Atomic displacement parameters (Å^2^)

**Table:**

	*U*^11^	*U*^22^	*U*^33^	*U*^12^	*U*^13^	*U*^23^
V1	0.01255 (12)	0.01250 (12)	0.01468 (12)	−0.00034 (9)	−0.00089 (8)	0.00034 (8)
O1	0.0192 (5)	0.0202 (5)	0.0224 (5)	0.0020 (4)	0.0010 (4)	−0.0016 (4)
O2	0.0180 (5)	0.0185 (5)	0.0191 (5)	−0.0031 (4)	−0.0018 (4)	0.0043 (4)
O3	0.0132 (5)	0.0166 (5)	0.0196 (5)	−0.0025 (4)	−0.0022 (4)	0.0028 (4)
O4	0.0155 (5)	0.0173 (5)	0.0190 (5)	0.0005 (4)	−0.0022 (4)	−0.0022 (4)
O5	0.0162 (5)	0.0176 (5)	0.0173 (5)	−0.0002 (4)	−0.0031 (4)	−0.0015 (4)
O6	0.0245 (6)	0.0265 (6)	0.0216 (5)	−0.0115 (5)	−0.0014 (4)	0.0063 (4)
N1	0.0151 (6)	0.0142 (5)	0.0163 (6)	−0.0009 (5)	−0.0007 (4)	0.0001 (4)
C1	0.0189 (7)	0.0172 (7)	0.0199 (7)	−0.0017 (6)	0.0035 (6)	0.0020 (5)
C2	0.0164 (7)	0.0199 (7)	0.0230 (7)	−0.0051 (6)	0.0019 (6)	0.0003 (6)
C3	0.0132 (6)	0.0170 (6)	0.0182 (7)	0.0004 (5)	0.0013 (5)	−0.0024 (5)
C4	0.0160 (7)	0.0246 (8)	0.0250 (8)	−0.0026 (6)	−0.0047 (6)	−0.0006 (6)
C5	0.0366 (10)	0.0311 (9)	0.0315 (9)	−0.0130 (8)	−0.0041 (7)	0.0151 (7)
C6	0.0172 (7)	0.0167 (7)	0.0162 (6)	0.0002 (5)	0.0022 (5)	0.0026 (5)
C7	0.0129 (7)	0.0282 (8)	0.0233 (7)	0.0017 (6)	−0.0003 (6)	−0.0023 (6)
C8	0.0147 (7)	0.0217 (7)	0.0207 (7)	−0.0014 (6)	−0.0029 (5)	0.0015 (6)
C9	0.0224 (8)	0.0238 (8)	0.0218 (7)	0.0028 (6)	0.0029 (6)	−0.0042 (6)
C10	0.0188 (8)	0.0435 (11)	0.0320 (9)	0.0003 (7)	−0.0087 (7)	−0.0111 (8)
C11	0.0186 (7)	0.0173 (7)	0.0165 (7)	−0.0031 (6)	−0.0035 (5)	−0.0013 (5)
C12	0.0169 (7)	0.0176 (7)	0.0201 (7)	−0.0046 (6)	−0.0011 (5)	−0.0022 (5)
C13	0.0174 (7)	0.0162 (6)	0.0178 (7)	−0.0015 (5)	0.0025 (5)	0.0007 (5)
C14	0.0166 (7)	0.0202 (7)	0.0160 (6)	−0.0022 (6)	−0.0020 (5)	0.0003 (5)
C15	0.0159 (7)	0.0180 (7)	0.0176 (7)	−0.0035 (6)	−0.0023 (5)	−0.0013 (5)
C16	0.0308 (9)	0.0289 (9)	0.0271 (8)	−0.0160 (7)	0.0030 (7)	0.0037 (7)

### Bis(acetylacetonato-κ2O,O')(4-methoxypyridine-κN)oxidovanadium(IV) (compound3) Geometric parameters (Å, º)

**Table:**

V1—O1	1.6035 (11)	C5—H5C	0.9800
V1—O3	1.9975 (10)	C6—C7	1.406 (2)
V1—O2	2.0110 (11)	C6—C9	1.511 (2)
V1—O5	2.0115 (10)	C7—C8	1.386 (2)
V1—N1	2.1440 (12)	C7—H7	0.9500
V1—O4	2.1767 (11)	C8—C10	1.508 (2)
O2—C1	1.2786 (18)	C9—H9A	0.9800
O3—C3	1.2770 (17)	C9—H9B	0.9800
O4—C6	1.2647 (18)	C9—H9C	0.9800
O5—C8	1.2835 (18)	C10—H10A	0.9800
O6—C13	1.3494 (17)	C10—H10B	0.9800
O6—C16	1.4342 (19)	C10—H10C	0.9800
N1—C11	1.3442 (18)	C11—C12	1.384 (2)
N1—C15	1.3541 (18)	C11—H11	0.9500
C1—C2	1.396 (2)	C12—C13	1.391 (2)
C1—C5	1.503 (2)	C12—H12	0.9500
C2—C3	1.397 (2)	C13—C14	1.397 (2)
C2—H2	0.9500	C14—C15	1.371 (2)
C3—C4	1.505 (2)	C14—H14	0.9500
C4—H4A	0.9800	C15—H15	0.9500
C4—H4B	0.9800	C16—H16A	0.9800
C4—H4C	0.9800	C16—H16B	0.9800
C5—H5A	0.9800	C16—H16C	0.9800
C5—H5B	0.9800		
			
O1—V1—O3	98.71 (5)	H5B—C5—H5C	109.5
O1—V1—O2	99.54 (5)	O4—C6—C7	124.29 (14)
O3—V1—O2	88.10 (4)	O4—C6—C9	117.52 (13)
O1—V1—O5	94.97 (5)	C7—C6—C9	118.18 (13)
O3—V1—O5	166.27 (4)	C8—C7—C6	123.87 (14)
O2—V1—O5	90.78 (4)	C8—C7—H7	118.1
O1—V1—N1	94.97 (5)	C6—C7—H7	118.1
O3—V1—N1	87.44 (4)	O5—C8—C7	125.57 (13)
O2—V1—N1	165.31 (5)	O5—C8—C10	115.03 (14)
O5—V1—N1	90.24 (5)	C7—C8—C10	119.40 (14)
O1—V1—O4	176.32 (5)	C6—C9—H9A	109.5
O3—V1—O4	83.48 (4)	C6—C9—H9B	109.5
O2—V1—O4	83.45 (4)	H9A—C9—H9B	109.5
O5—V1—O4	82.80 (4)	C6—C9—H9C	109.5
N1—V1—O4	82.13 (4)	H9A—C9—H9C	109.5
C1—O2—V1	128.18 (9)	H9B—C9—H9C	109.5
C3—O3—V1	129.31 (9)	C8—C10—H10A	109.5
C6—O4—V1	129.46 (9)	C8—C10—H10B	109.5
C8—O5—V1	132.83 (9)	H10A—C10—H10B	109.5
C13—O6—C16	117.11 (12)	C8—C10—H10C	109.5
C11—N1—C15	116.68 (12)	H10A—C10—H10C	109.5
C11—N1—V1	121.24 (10)	H10B—C10—H10C	109.5
C15—N1—V1	121.92 (10)	N1—C11—C12	124.07 (13)
O2—C1—C2	125.53 (13)	N1—C11—H11	118.0
O2—C1—C5	115.67 (13)	C12—C11—H11	118.0
C2—C1—C5	118.80 (14)	C11—C12—C13	118.10 (13)
C1—C2—C3	124.06 (14)	C11—C12—H12	121.0
C1—C2—H2	118.0	C13—C12—H12	121.0
C3—C2—H2	118.0	O6—C13—C12	124.98 (13)
O3—C3—C2	124.77 (13)	O6—C13—C14	116.26 (13)
O3—C3—C4	115.17 (13)	C12—C13—C14	118.76 (13)
C2—C3—C4	120.05 (13)	C15—C14—C13	118.85 (13)
C3—C4—H4A	109.5	C15—C14—H14	120.6
C3—C4—H4B	109.5	C13—C14—H14	120.6
H4A—C4—H4B	109.5	N1—C15—C14	123.52 (13)
C3—C4—H4C	109.5	N1—C15—H15	118.2
H4A—C4—H4C	109.5	C14—C15—H15	118.2
H4B—C4—H4C	109.5	O6—C16—H16A	109.5
C1—C5—H5A	109.5	O6—C16—H16B	109.5
C1—C5—H5B	109.5	H16A—C16—H16B	109.5
H5A—C5—H5B	109.5	O6—C16—H16C	109.5
C1—C5—H5C	109.5	H16A—C16—H16C	109.5
H5A—C5—H5C	109.5	H16B—C16—H16C	109.5
			
V1—O2—C1—C2	2.0 (2)	C6—C7—C8—O5	3.7 (3)
V1—O2—C1—C5	−177.67 (11)	C6—C7—C8—C10	−175.99 (16)
O2—C1—C2—C3	−1.8 (3)	C15—N1—C11—C12	−0.6 (2)
C5—C1—C2—C3	177.87 (15)	V1—N1—C11—C12	174.86 (11)
V1—O3—C3—C2	2.2 (2)	N1—C11—C12—C13	−0.9 (2)
V1—O3—C3—C4	−177.27 (10)	C16—O6—C13—C12	−2.3 (2)
C1—C2—C3—O3	−0.4 (2)	C16—O6—C13—C14	177.13 (14)
C1—C2—C3—C4	179.04 (14)	C11—C12—C13—O6	−179.03 (14)
V1—O4—C6—C7	−2.3 (2)	C11—C12—C13—C14	1.6 (2)
V1—O4—C6—C9	179.17 (9)	O6—C13—C14—C15	179.74 (13)
O4—C6—C7—C8	−6.1 (3)	C12—C13—C14—C15	−0.8 (2)
C9—C6—C7—C8	172.47 (15)	C11—N1—C15—C14	1.4 (2)
V1—O5—C8—C7	8.0 (2)	V1—N1—C15—C14	−173.97 (11)
V1—O5—C8—C10	−172.33 (11)	C13—C14—C15—N1	−0.7 (2)

### Bis(acetylacetonato-κ2O,O')(4-methoxypyridine-κN)oxidovanadium(IV) (compound3) Hydrogen-bond geometry (Å, º)

**Table:**

*D*—H···*A*	*D*—H	H···*A*	*D*···*A*	*D*—H···*A*
C2—H2···O1^i^	0.95	2.57	3.488 (2)	163
C12—H12···O2^ii^	0.95	2.37	3.2567 (18)	156
C14—H14···O4^iii^	0.95	2.57	3.2462 (18)	129

Symmetry codes: (i) −*x*+3/2, *y*+1/2, −*z*+1/2; (ii) −*x*+1/2, *y*−1/2, −*z*+1/2; (iii) −*x*+1, −*y*+1, −*z*+1.
